# Galactose-Deficient IgA1 Deposits in Clear Cell Renal Cell Carcinoma-Related Henoch–Schönlein Purpura Nephritis

**DOI:** 10.1155/2020/8828336

**Published:** 2020-08-22

**Authors:** Yuhong Zhao, Youngki Kim, Milind Junghare, Viral Vakil, Xuesong Su, Faqian Li, Lihong Bu

**Affiliations:** ^1^Department of Nephrology, Central Hospital Affiliated to Shenyang Medical College, Shenyang, Liaoning 110024, China; ^2^Department of Pediatrics, Division of Pediatric Nephrology, University of Minnesota, Minneapolis, MN 55455, USA; ^3^Department of Renal Disease and Hypertension, University of Minnesota, Minneapolis, MN 55455, USA; ^4^Department of Nephrology, Shengjing Hospital of China Medical University, Shenyang, Liaoning 110004, China; ^5^Department of Laboratory Medicine and Pathology, University of Minnesota, Minneapolis, MN 55455, USA

## Abstract

Recent studies suggest that galactose-deficient IgA1 (Gd-IgA1) plays a role in the pathogenesis of primary IgA nephropathy (IgAN) and Henoch–Schönlein purpura nephritis (HSPN). Furthermore, immunostaining of KM55, an antibody that identifies Gd-IgA1, may be helpful to differentiate primary IgAN and HSPN from secondary causes of glomerular IgA deposition. We report sequential kidney biopsies of a malignancy-associated HSPN, showing intense glomerular mesangial IgA deposition at the initial kidney biopsy and dramatic decrease in disappearance of glomerular deposits after tumor removal. We demonstrate that the glomerular IgA deposition contains Gd-IgA1, detected by immunostaining of KM55, with similar distribution and intensity to IgA. This suggests that renal Gd-IgA1 deposition may play a role in the pathogenesis of malignancy-associated HSPN.

## 1. Introduction

Henoch–Schönlein purpura (HSP), also referred as immunoglobulin A vasculitis (IgAV), is the most common systemic vasculitis involving the small blood vessels in pediatric population with kidney involvement present in 30–50% [1]. Similar to IgA nephropathy (IgAN), Henoch–Schönlein purpura nephritis (HSPN) is characterized by the deposition of IgA dominant or codominant immune complex in the glomerular mesangial and capillary wall regions. The glomerular IgA deposition in IgAN is predominantly of the IgA1 subclass [2]. Galactose-deficient IgA1 (Gd-IgA1) has been identified to be a key element in the pathogenesis of IgAN with glomerular deposition of autoantibodies recognizing overproduced, aberrantly glycosylated Gd-IgA1 [3]. KM55, a Gd-IgA1-specific monoclonal antibody generated by immunizing rats with the human Gd-IgA1 hinge region peptide, has been shown to recognize Gd-IgA1 in renal biopsy tissue on formalin-fixed paraffin-embedded sections [4, 5]. Recent study shows KM55 is specifically detected in primary IgAN and HSPN, but not in other examined renal diseases, such as lupus nephritis, cirrhosis, or hepatitis C-related glomerular disease [6]. These findings suggest IgAN and HSPN share a common feature involving Gd-IgA1 in pathogenesis [6]. However, the specificity of KM55 for primary IgAN and HSPN was questioned in a more recent study demonstrating KM55 staining in incidental IgA deposition, secondary IgAN, and staphylococcal infection-associated glomerulonephritis, albeit with weaker staining than in primary IgAN [7].

Unlike HSPN in children, clinical presentation of HSPN in adults is frequently severe [8] and associated with malignancies in up to 28% [9] of cases, including hematolymphoid neoplasm and solid tumor from primary organ of respiratory, digestive, neurologic, and urologic systems [10, 11]. The pathophysiology of malignancy-associated HSPN remains speculative. Proposed mechanisms include abnormal production of antibodies directed against tumor neoantigens leading to the formation of immune complexes and similarities between tumor antigens and endothelial cell antigens. It is unknown whether Gd-IgA1 plays a role in the pathogenesis of malignancy-associated HSPN.

Here, we report detailed clinical pathological findings and immunostaining of KM55 on sequential kidney biopsies of a 35-year-old man with a clear cell renal cell carcinoma- (CCRCC-) associated HSPN.

## 2. Case Presentation

### 2.1. Initial Clinical History, Laboratory Data, and Pathology Findings

A 35-year-old man with a past history of schizoaffective disorder controlled by medication was admitted with a bilateral, palpable, nontender rash on his legs, leg stiffness, and leg pain for 24–36 hours. The patient complained of nausea, constipation, and worsening abdominal pain. No fevers, chills, diarrhea, difficulty in urinating, shortness of breath, or cough were noted. There was no personal or family history of rheumatologic disease or hypertension. There was no history of recent ill contacts, travel, wilderness exposure, or tick bites. The pertinent laboratory results were as follows: serum creatinine, 0.75 mg/dL (Table 1) and serum albumin, 3.2 g/dL. Urinalysis (UA) revealed a protein albumin urine of 100 mg/dL and 7 RBCs/hpf. Serological testing was negative for antinuclear antibodies (ANAs), double-stranded DNA (dsDNA), and antineutrophil cytoplasmic antibodies (ANCA). Complement levels and rheumatoid factor were normal. Renal ultrasound showed a 6.0 cm complex lesion in the lower pole of the right kidney.

**Figure 1:**
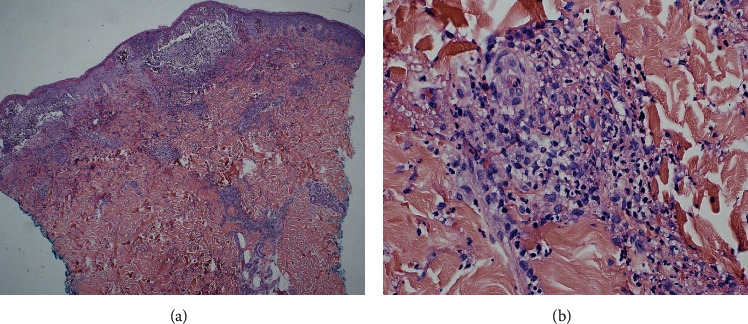
A punch biopsy of skin involved by a brisk acute small vessel neutrophilic vasculitis that extends from the papillary to midreticular dermis. There is prominent associated fibrinoid necrosis of vessel wall, leukocytoclasis, purpura, and epidermal degeneration. Original magnification: 4X in (a) and 40X in (b).

**Figure 2:**
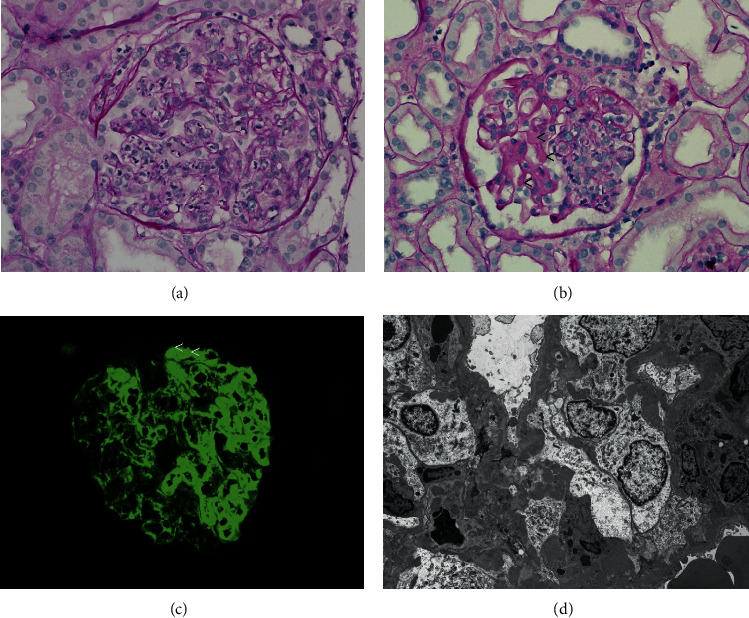
Findings of initial kidney biopsy: (a) a representative glomerulus with global endocapillary proliferation on periodic acid-Schiff stain; (b) a glomerulus with segmental mesangial and endocapillary proliferation (right side) and intracapillary pseudothrombi (<); (c) immunofluorescence staining of IgA shows global granular mesangial and capillary wall staining and a few intracapillary pseudothrombi (<); (d) electron microscopy shows many electron dense mesangial and subendothelial deposits with severe foot process effacement. Original magnification: 400X in (a–c) and 5000X in (d).

**Figure 3:**
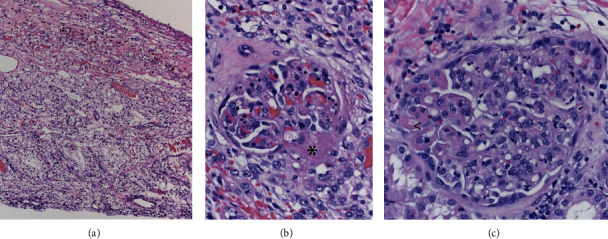
Clear cell renal cell carcinoma, low grade of partial nephrectomy (a). In adjacent nonneoplastic kidney, several glomeruli show segmental to global endocapillary proliferation with segmental necrotizing lesion (*∗*) in (b) and rare glomeruli show large glomerular deposits (<) in (c). Original magnification: 40X in (a) and 400X in (b) and (c).

Two days after admission, a skin biopsy of the lower shin was performed, showing leukocytoclastic vasculitis (Figure 1) with intravascular deposition of IgA and C3 (not shown), consistent with HSP. The patient was started with oral prednisone at a dose of 60 mg daily with improvement of the rash, leg pain, and abdominal pain. In the next week, the patient's symptoms worsened with oliguria, rapid increase in serum creatinine (from 0.75 to 5.14 mg/dL) (Table 1), and nephrotic-range proteinuria (8.9 g/g Cr). UA showed 13 RBCs/hpf while on pulse methylprednisolone (500 mg) intravenously (IV) for 3 days (Table 1). Dialysis was initiated, and mycophenolate mofetil (MMF) (1 g twice daily by mouth) was added. A kidney biopsy showed diffuse global endocapillary and focal segmental mesangial proliferative glomerulonephritis with IgA-dominant mesangial and capillary wall deposition, consistent with active HSPN (Figure 2). With the consideration of paraneoplastic syndrome, an urgent partial nephrectomy was performed. A clear cell renal cell carcinoma (CCRCC) pT1b was diagnosed. A few crescentic lesions were identified in the nonneoplastic kidney of the partial nephrectomy specimen (Figure 3). Renal function improved, and dialysis was discontinued seven days following the nephrectomy. The patient was discharged on the postoperative day eight on a regimen of mycophenolate and prednisone.

### 2.2. Follow-Up and Sequential Kidney Biopsy Findings

**Figure 4:**
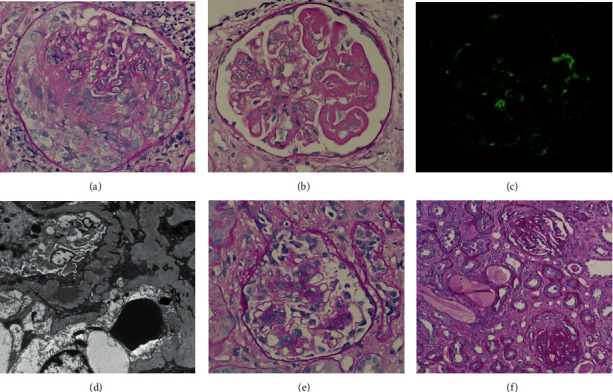
Findings of the second and third kidney biopsies, at three weeks (a, b) and five months (c–f) after surgical resection of clear cell renal cell carcinoma, respectively. Sequential biopsies demonstrate progression and transition from active lesions (a, b) to sclerosing lesions (e, f), and dramatic clearance of glomerular IgA deposits following tumor resection (c, d). (a) A representative glomerulus shows endocapillary hypercellularity and a nearly circumferential cellular crescent with necrosis. (b) Segmental large subendothelial deposits (right half of the glomerulus) are visible by light microscopy. (c) Immunofluorescence staining of IgA shows only modest glomerular mesangial and capillary wall IgA deposition. (d) Electron microscopy shows a small number of electron dense deposits mainly in mesangium. (e) Nonsclerosed glomeruli show only mild mesangial hypercellularity without endocapillary hypercellularity. (f) Representative glomeruli with fibrous crescents, one globally sclerotic (the lower glomerulus) and the other segmentally scarred (the upper glomerulus). The third biopsy shows significant chronic changes with approximately 35% global glomerulosclerosis and additional 35% glomeruli with fibrous crescents and segmental sclerosis. There is severe tubulointerstitial scarring. Original magnification: 400X in (a–c) and (e), 8000X in (d), and 200X in (f).

Two weeks later, the patient was admitted for shortness of breath with an increase in creatinine (from 2.58 mg/dL to 4.76 mg/dL) (Table 1) and oliguria caused by a leak in his nephrectomy wound. A second renal biopsy was performed at three weeks after surgical removal of the kidney tumor. The biopsy revealed diffuse cellular and fibrocellular crescents and minimal IgA deposition along glomerular capillary wall (Table 1; Figures 4(a) and 4(b)). Therapeutic plasma exchange (PLEX) was performed every other day for a total of 2 weeks, along with IV cyclophosphamide at 15 mg/kg x1 and continuation of the prednisone and MMF. Several days after discharge, the patient had pancytopenia, hematochezia, and epistaxis. MMF was discontinued due to concern of side effect, while prednisone and cyclophosphamide were continued with PLEX. Over the next two weeks, there was no obvious improvement in renal function. Fluid retention was controlled by renal replacement therapy. The steroid was tapered, and cytoxan was given orally instead of intravenously. During the next 4 months, clinical manifestation and laboratory tests of the patient remained stable (Table 1).

To further clarify the kidney disease etiology, determine the level of chronic kidney damage, and assess disease activity while continuing to treat with cytotoxic therapies, a third renal biopsy was performed at five months after surgical resection of the kidney tumor. The renal biopsy showed only chronic changes with fibrous crescents and negligible IgA deposition in nonsclerotic glomeruli (Figures 4(c)–4(f)). Therefore, cytoxan was discontinued, and prednisone was tapered off over the next 21 days, with continued renal replacement therapy.

### 2.3. Gd-IgA1 Immunohistochemical Staining on Kidney Biopsies

**Figure 5:**
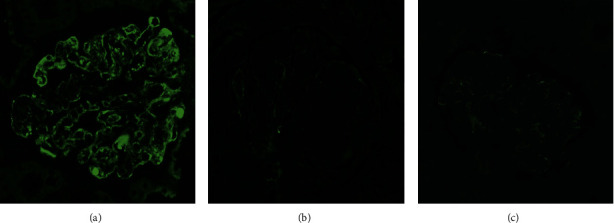
Immunofluorescence staining of KM55 shows global, granular, mesangial, and capillary wall as well as intracapillary pseudothrombi staining on initial kidney biopsy (a) and diminished/minimal glomerular capillary wall staining on sequential kidney biopsies three weeks (b) and five months (c) after surgical resection of clear cell renal cell carcinoma. Original magnification: 400X in all.

Gd-IgA1 (anti-human Gd-IgA1 (KM55) rat IgG monoclonal antibody, Immuno-Biological Laboratories Co., Ltd, at a concentration of 50 *μ*g/mL) was stained for all three kidney biopsies, one prior to partial nephrectomy and two after surgical removal of CCRCC. KM55 displayed similar staining pattern and intensity to IgA in all biopsies (Figure 5). Diffuse global granular capillary wall staining and segmental granular mesangial staining for Gd-IgA1, similar to IgA, were observed on the first biopsy, while dramatically decreased Gd-IgA1 and IgA staining along the capillary wall and in mesangium were seen on the follow-up kidney biopsies (Figure 5).

## 3. Discussion

Malignancy-associated HSPN usually affects patients older than 40 years of age (range from 29 to 79), with a male predominance [9]. The majority of the neoplasms (80%) are detected at the time of or within 1 year after the occurrence of HSP, and 67% of the malignancies are solid tumors [9]. The renal manifestations of HSPN can range from microscopic hematuria and proteinuria only to a rapidly progressive glomerulonephritis (RPGN). Of those who undergo a kidney biopsy, 95% show IgA deposition in the mesangial area [12–22]. The most frequent lesion in renal biopsies is proliferative endocapillary glomerular nephritis [11]. The outcome of HSPN in adults is relatively poor, and the most frequent cause of death was neoplasia (up to 27% of death) [10–12, 14, 16, 18, 21] although significant improvement of renal function was reported in some cases with treatment of the cancer and corticosteroids for HSPN [13–15, 22]. The independent prognostic factors of renal outcome in patients with HSPN are proteinuria level, initial renal failure, and the histologic quantification of interstitial fibrosis and glomerular sclerosis [11].

Our patient's clinical characteristics and biopsy findings are in concordance with the reported cases in the literature. The disappearance of glomerular IgA deposits soon after tumor removal supports that the patient's HSPN is associated with the patient's concurrent kidney cancer. The disappearance of mesangial IgA deposits on repeat biopsies has been rarely reported in children [23] and adults [24]; however, the disappearance is observed after prolonged combination therapy, two years and 77 months on average for pediatric and adult patients, respectively. Despite tumor removal and immunosuppression treatment, our patient's renal function deteriorated and the patient was on maintenance dialysis, as reported in other cases [17, 21].

Gd-IgA1 plays an important role in the pathogenesis of IgAN. Gd-IgA1 is reported to increase in blood circulation and/or be deposited in renal mesangium in patients with primary IgAN and/or HSPN [25–28]. The data are sparse and controversial on whether immunostaining of KM-55, a Gd-IgA1-specific monoclonal antibody, is specific for patients with primary IgAN and HSPN, but not secondary form [6, 7, 29]. Here, we demonstrate glomerular KM55 staining with a similar pattern and intensity to IgA in this malignancy-associated HSPN, a secondary form of IgA deposition. These data show that KM55 staining may not be helpful in distinguishing primary from secondary HSPN. Furthermore, the KM55 staining in the sequential kidney biopsies suggests the production of autoantibodies against Gd-IgA1 molecules might be one of the pathophysiologies of malignancy-associated HSPN.

## 4. Conclusions

Here, we report KM55 staining on three sequential kidney biopsies from a 35-year-old man with HSPN and a concurrent kidney carcinoma. Positive glomerular staining of KM-55, an antibody that identifies Gd-IgA1, in this malignancy-associated HSPN was seen with a similar staining pattern and intensity to IgA on the initial kidney biopsy. Disappearance of glomerular mesangial IgA and Gd-IgA1 deposits was observed on two sequential kidney biopsies at three weeks and five months after surgical resection of the kidney tumor. The findings suggest Gd-IgA1, not specific to primary IgAN and HSPN, may play a role in the pathogenesis of malignancy-associated HSPN.

## Figures and Tables

**Table 1:** Laboratory results before each kidney biopsy and after tumor resection.

Variables	Baseline (at presentation)	Before the 1^st^ kidney biopsy	Three weeks after tumor resection (before the 2^nd^ kidney biopsy)	Five months after tumor resection (before the 3^rd^ kidney biopsy)	Reference range
Sodium (mmol/L)	140	134	144	138	133–144
Potassium (mmol/L)	4.5	4.0	3.7	4.6	3.4–5.3
Chloride (mmol/L)	105	101	115	104	94–109
Carbon dioxide (mmol/L)	24	20	15	24	20–32
Calcium (mg/dL)	9.4	7.5	7.1	8.2	8.5–10.1
Phosphorus (mg/dL)	3.8	5.6	7.4	3.9	2.4–4.5
BUN (mg/dL)	12	62	58	32	7–30
Cr (mg/dL)	0.75	5.14	4.76	4.89	0.66–1.25
Protein, total (g/dL)	7.2	5.2	3.8	4.7	6.8–8.8
Albumin (g/dL)	3.6	1.6	1.0	1.4	3.4–5.0
Protein, total urine (g/gr Cr.)	−	8.91	9.19	16.99	0–0.2
Glucose (mg/dL)	98	84	80	101	70–99
WBC (10^9^/L)	10.9	18.7	5.8	2.5	4.0–11.0
Hemoglobin (g/dL)	14.6	10.3	7.7	8.7	13.3–17.7
Platelets (10^9^/L)	317	204	335	123	150–450
ANA	—	<1.0	—	—	<1.0
dsDNA (IU/mL)	—	2	—	—	<10
ANCA	—	<1 : 20	—	—	—
Cryoglobulin (%)	—	—	Neg	—	(NEG%)
C3 (mg/dL)	—	130	110	—	76–169
C4 (mg/dL)	—	15	23	—	15–50
IgA (mg/dL)	—	118	—	—	70–380
IgG (mg/dL)	—	479	—	—	695–1620
IgM (mg/dL)	—	25	—	—	60–265
Myeloperoxidase Ab, IgG (AI)	—	0.2	0.2	—	0.0–0.9
Proteinase 3Ab, IgG (AI)	—	0.2	0.2	—	0.0–0.9
